# Podocalyxin in Normal Tissue and Epithelial Cancer

**DOI:** 10.3390/cancers13122863

**Published:** 2021-06-08

**Authors:** Ngoc Le Tran, Yao Wang, Guiying Nie

**Affiliations:** Implantation and Pregnancy Research Laboratory, School of Health and Biomedical Sciences, RMIT University, Bundoora 3083, Australia; s3861978@student.rmit.edu.au (N.L.T.); yao.wang2@rmit.edu.au (Y.W.)

**Keywords:** podocalyxin, PODXL, epithelial cancer, metastasis, EMT, tumour budding, migration, invasion, extravasation, immune evasion, chemoresistance

## Abstract

**Simple Summary:**

Malignancies derived from epithelial cells account for over 90% of all human cancers. Several aspects of cancer progression such as metastasis, immune evasion, and resistance to chemotherapy are often associated with poor prognosis and unfavourable patient outcomes due to limited therapeutic options. Therefore, the discovery of new biomarkers and treatment targets is essential in improving patient prognosis. Podocalyxin, a membrane protein of the CD34 family, has recently gained recognition as a potential diagnostic and prognostic biomarker, as well as a potential target for chemoresistance. This review summarises the current knowledge of podocalyxin in normal tissues and epithelial cancers, highlighting its potential utility in cancer management.

**Abstract:**

Podocalyxin (PODXL), a glycosylated cell surface sialomucin of the CD34 family, is normally expressed in kidney podocytes, vascular endothelial cells, hematopoietic progenitors, mesothelium, as well as a subset of neurons. In the kidney, PODXL functions primarily as an antiadhesive molecule in podocyte epithelial cells, regulating adhesion and cell morphology, and playing an essential role in the development and function of the organ. Outside the kidney, PODXL plays subtle roles in tissue remodelling and development. Furthermore, many cancers, especially those that originated from the epithelium, have been reported to overexpress PODXL. Collective evidence suggests that PODXL overexpression is linked to poor prognosis, more aggressive tumour progression, unfavourable treatment outcomes, and possibly chemoresistance. This review summarises our current knowledge of PODXL in normal tissue function and epithelial cancer, with a particular focus on its underlying roles in cancer metastasis, likely involvement in chemoresistance, and potential use as a diagnostic and prognostic biomarker.

## 1. Introduction

The epithelium consists of a layer of apical-basally polarised cells that line the cavity and surfaces of organs throughout the body. Depending on their location, epithelial cells may serve several functions including barrier protection, secretion, and absorption [1]. The epithelium is the most common site for the development of cancers, accounting for approximately 90% of all human malignancies [2]. The metastatic potential of epithelial tumours to secondary sites heralds advanced disease which is highly correlated with poor prognosis and increased mortality [3]. Therefore, identifying biomarkers that detect epithelial tumours likely to metastasise, is key to improving patient outcomes and survival.

PODXL is a type 1 transmembrane sialomucin belonging to the CD34 family and was originally identified as an apical membrane protein predominantly expressed in kidney glomeruli [4]. PODXL is now shown to also be expressed on the surface of vascular endothelial cells [5], megakaryocytes and platelets [6], mesothelial cells [4], hematopoietic progenitors [7], and a subset of neurons [8]. In epithelial cells, PODXL interacts with adaptor proteins that are implicated in actin binding, protein trafficking, and signalling, allowing PODXL to play a pivotal role in many physiological processes including embryonic development, inflammatory responses, and cancer metastasis [9,10]. While PODXL is detected in normal tissues of the kidney, breast, liver, pancreas, and endometrium [11,12], overexpression of PODXL has been associated with poor prognosis and outcomes of several epithelial cancers, including breast and ovarian carcinomas [13,14]. The function of PODXL in regulating cell adhesion has been attributed to its role in promoting aggressive epithelial cancer phenotypes, as well as enhanced cancer cell growth, invasion, migration, and metastasis [13,15,16,17,18]. Thus, PODXL may be a potential biomarker for predicting patient outcomes and identifying subgroups with higher risks of developing aggressive cancer phenotypes [15,19]. Moreover, understanding the involvement of PODXL in cancer progression may offer further insight into the mechanisms underlying the metastasis process for developing better treatment.

This review aims to provide an up-to-date summary of the current literature on PODXL in normal tissues and cancerous epithelial cells. We particularly examine the mechanisms of PODXL in cancer progression and its potential as a biomarker in the diagnosis, prognosis, and treatment of epithelial cancers.

## 2. An Overview of PODXL in Normal Development and Function

**Figure 1:**
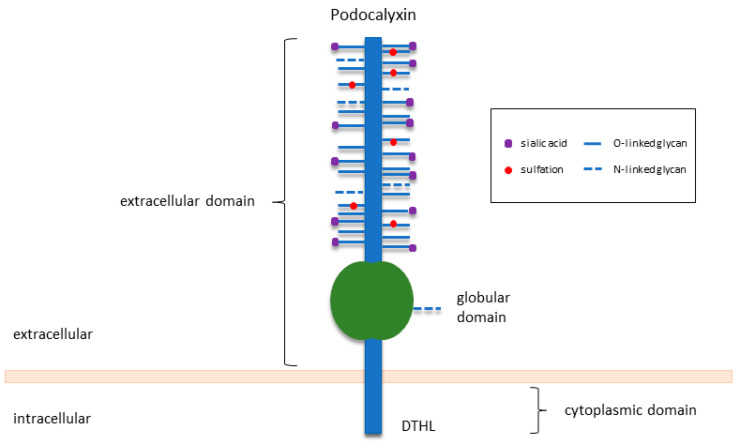
Structure of PODXL. Adapted from McNagny and Hughes [20].

PODXL, also known as podocalyxin-like protein-1, PCLP1 or PCX, is a type I transmembrane protein of the sialomucin family of CD34. Human PODXL is a 558-amino-acid long protein that is heavily modified post-translationally to yield an end product of 140–200 kDa in molecular weight [20]. Preserving the basic structure of family members CD34 and endoglycan, PODXL consists of a highly conserved cytoplasmic domain with a C-terminal PDZ binding region (DTHL motif), a single-pass transmembrane domain, and an extensively O-glycosylated and sialylated extracellular domain (Figure 1) [21,22,23]. These glycosylation and sulfation modifications give rise to a highly negatively charged structure, which is essential for the maintenance of open filtration assemblies between neighbouring podocytes in the kidney [4].

In general, PODXL mRNA expression is enhanced by the Wilms’ tumour 1 (WT1) tumour suppressor and transcription factor specificity protein 1 (Sp1), but it is repressed by the transcription factor P53 (TP53) and the integrin-associated adaptor protein PINCH1 (LIMS1) [24,25,26,27]. P53 functions as a tumour suppressor by activating multiple cellular processes including DNA repair, cell cycle arrest, and apoptosis [28]. Mutations in P53 are one of the most well-known and frequent genomic alterations in human cancers, and expression of mutated P53 has been linked to poor patient outcomes in a variety of cancers [29]. Therefore, mutations in the upstream biological pathways of PODXL, such as WT1 and P53, can induce changes in PODXL expression that may contribute to tumourigenesis [26].

### 2.1. PODXL Is Essential for Kidney Development and Function

PODXL is highly expressed on the apical surface of specialised glomerular epithelial cells called podocytes, which are essential for kidney development and function [4]. Podocytes undergo a series of extensive morphological changes during kidney development, coinciding with the opening of tight junctions and the appearance of urinary spaces that provide the pathway for glomerular filtration [30]. Together with capillary loops, fenestrated endothelial cells, and the glomerular basement membrane, podocytes form glomeruli which are responsible for blood filtration and urine formation in the kidney [23,31]. The apical surface of podocytes is coated with a sialic acid-rich glycocalyx known as the glomerular epithelial polyanion, which is highly negatively charged and composed mainly of PODXL [32]. PODXL is expressed along the apical surface of the podocyte cell body and foot processes in coupling with the appearance of open intercellular spaces [33]. While originally postulated to maintain the opening of filtration slits through its charge repulsion properties, PODXL was later demonstrated to also have antiadhesive properties that are responsible for keeping the intercellular spaces open [34]. Removal of sialic acids and the associated negative charges on the extracellular domain of PODXL eliminates its antiadhesive property and causes the filtration slits to collapse [34,35]. The antiadhesive activity of PODXL thus plays a crucial role in physically separating the podocyte foot processes to enable their urinary filtering function. Abnormal PODXL expression leads to loss of podocyte integrity, disruption of the glomerulus organisation, and development of kidney diseases [36]. Consequently, detection of urinary PODXL resulting from podocyte damage and excretion has been correlated with diabetic nephropathy and renal injury [37]. Mice lacking PODXL show a loss of podocyte integrity, failure of glomerular foot formation, and collapse of the filtration slits [11]. PODXL knockout mice, while producing normal numbers of podocyte precursors, fail to form highly interdigitated foot processes, and instead retain tight cell junctions between immature neighbouring podocytes [11], and ultimately suffer from kidney failure and perinatal lethality [4,11]. These studies highlight the importance of PODXL as a charge-selective barrier and antiadhesive mucin in the maintenance of podocyte morphology and glomerular filtration function.

### 2.2. PODXL Also Plays Subtle Roles in Other Tissues

Outside the kidney, PODXL has more subtle roles in aiding tissue remodelling and development [20]. During embryonic development, PODXL in mesothelial cells is required for retracting the embryonic gut from the umbilical cord, and loss of PODXL leads to gut herniation or omphalocele in 30% of mice [11]. In adult mice and humans, PODXL is expressed in vascular endothelial cells, hematopoietic stem cells, and a subset of neurons [5,11]. It is also present in all haematopoietically active tissues, hematopoietic progenitor cells, and erythroblasts [38,39]. During early development, PODXL is expressed on primitive murine erythroid cells (embryonic day 7–12), marking both circulating erythroblasts and their progenitors, while in adult mice, PODXL expression is restricted to erythroid progenitors during erythropoiesis stress conditions such as anaemia [40,41,42]. In rats, PODXL expression is more restricted to activated platelets and megakaryocytes [6].

As a universal marker for the vasculature, PODXL is well expressed on endothelial cells lining blood vessels such as the high endothelial venules (HEV) [5]. Similar to podocytes, the endothelium is endowed with a negatively charged sialoprotein surface covering which is largely made of PODXL [5]. PODXL in endothelial cells is reported to be pro-adhesive [43]. In HEV, PODXL mediates binding to the leukocyte adhesion molecules such as L-selectin, to facilitate lymphocyte migration and recruitment to sites of inflammation [44]. PODXL also critically participates in the formation of appropriate basolateral domains and barrier functions [45]. In the lung and blood–brain barrier (BBB), mice with conditioned deletion of PODXL in vascular endothelial cells (PODXL^-EC^) exhibit leakage of plasma from both the lung parenchyma and BBB [46]. Lung endothelial cells from these mice show increased expression of integrins and matrix proteins and a significantly impaired ability to spread on laminin, a component of the ECM, suggesting a role of PODXL in regulating endothelial cell–laminin basement membrane interactions [46]. Though displaying no major overt defects, PODXL^-EC^ mice show a moderate rise in lung vascular permeability, which, however, is greatly exacerbated in response to lipopolysaccharide (LPS)-induced inflammation [46]. Similarly, in the absence of PODXL, vascular endothelial cells of the central nervous system only exhibit a moderate increase in BBB permeability; however, the LPS-induced inflammation further compromises the condition, resulting in transient suppression of cerebral cortex activity [45]. Furthermore, PODXL is well expressed in human umbilical vein endothelial cells (HUVECs) [47]. When PODXL is knocked down in HUVECs, actin is disorganised, binding to the basal matrix is impaired, cell–cell interactions are weakened, and focal adhesions and adherens junctions are mis-localised [45]. Therefore, PODXL likely plays a critical role in promoting endothelial morphogenesis and maintaining the integrity of functional endothelial cell barriers in a matrix-dependent manner.

In the brain, PODXL is expressed in many regions with the highest level detected in the postnatal cerebral cortex and cerebellum [8]. During brain development, PODXL is expressed in proliferative regions, migratory pathways, and synapses. Despite this, the brain of PODXL-null mice retains a normal layered organisation, suggesting that PODXL is not required for neuronal proliferation or migration [48]. Likewise, the hematopoietic, vascular endothelial, and brain cells in PODXL knockout mice develop normally and do not display any obvious defects [11]. However, PODXL-null tissues express higher levels of CD34, suggesting that the upregulation of this family member may have compensated for the loss of PODXL [11].

In the human endometrium (the inner lining of the uterus), PODXL expression has been observed on the apical surface of epithelial and endothelial cells [12,49]. Recent studies have identified PODXL as a key negative regulator of endometrial epithelial receptivity for embryo implantation [12]. PODXL is expressed in both the luminal and glandular epithelium in the non-receptive state, but in the mid-secretory phase of the menstrual cycle when the endometrial becomes receptive for the embryo implantation, PODXL is downregulated selectively in the luminal epithelium, to which an embryo would first encounter to initiate implantation. When PODXL is overexpressed in Ishikawa cells, a widely used receptive human endometrial epithelial cell line, the ability of embryo mimics to attach to or penetrate through the Ishikawa monolayer is significantly inhibited [12]. These results suggest that PODXL inhibits implantation and that downregulation of PODXL in the luminal epithelium of the uterus is essential for developing endometrial receptivity. 

## 3. PODXL in Human Malignancies, Specifically in Epithelial Cancers

Since its first discovery as a stem cell marker in testicular cancer [50], PODXL has been detected in several solid human malignancies, including germ cell tumours [50], astrocytoma [51], haematological cancers [52,53], and numerous epithelial malignancies including cancers of the breast, kidney, colon or rectum, prostate, pancreatic ducts, urothelial bladder, ovary, lung, thyroid, oesophagus, oral squamous cells, uterus, and stomach (Table 1). In particular, high PODXL expression has been associated with high-grade tumours, aggressive tumour phenotypes, and poor prognosis in breast cancer [15,54], colorectal cancer [19,55,56,57], gastric cancer [58,59,60], hepatocellular carcinoma [54,61], lung cancer including small cell lung carcinoma [62], and lung adenocarcinoma [63]. Similar observations have also been made for oesophageal cancer [59], oral squamous cell carcinoma [64], ovarian cancer [14], pancreatic ductal adenocarcinoma [65,66,67,68], periampullary adenocarcinoma [67], renal cell carcinoma [69], undifferentiated thyroid carcinoma [49], urothelial bladder cancer [70,71], and uterine endometrioid adenocarcinoma [72]. High PODXL has also been associated with poor tumour differentiation [73] and estrogen- and progesterone-receptor-negative tumours [15,74].

PODXL mutation rather than overexpression is strongly linked to aggressive prostate cancer [75,76,77]. A locus on the human chromosome 7q32-33 where the PODXL gene is located exhibits a high frequency of allelic imbalance in prostate tumours, where several missense mutations and in-frame deletions within PODXL are associated with increased risk for developing more aggressive prostate cancer [16]. 

While metastatic cancers account for more than 90% of all cancer-related deaths, the mechanisms behind the phenomenon are unclear [80]. PODXL as a mediator for metastasis may provide a useful biomarker for assessing metastasis potential and prognosis in cancer patients. While the exact mechanisms of PODXL in tumourigenesis are not completely understood, studies in human cancer cell lines suggest that PODXL may directly contribute to tumour aggression by facilitating tumour migration, invasion, metastases, and chemoresistance (Table 2). Possible mechanisms of PODXL in promoting epithelial cancer are discussed below. 

### 3.1. PODXL Activates Intracellular Signalling Pathways to Facilitate Cancer Metastasis

**Figure 2:**
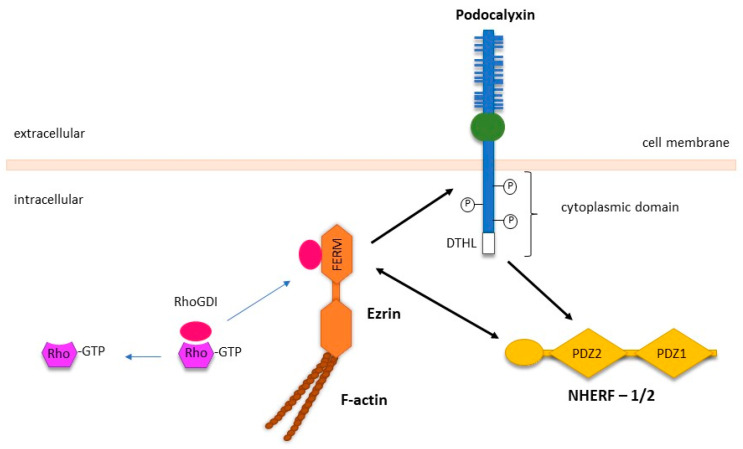
Interactions between PODXL and its intracellular binding partners’ ezrin and NHERF1/2. The intracellular domain of PODXL contains several phosphorylation sites and a C-terminal DTHL motif, which can directly interact with with the N-terminal FERM domain of ezrin. In addition, ezrin can interact with PODXL indirectly via the C-terminal of NHERF1/2, following the binding of the DTHL motif of PODXL to PDZ2 domain of NHERF1/2. Activation of the PODXL-NHERF-1/2-ezrin complex results in subsequent binding to the actin cytoskeleton. Sequestration of RhoGDI by ezrin releases Rho-GDP, which converts to Rho-GTP to enhance the activation of Rho-family GTPases. Specific protein-protein interactions are indicated by the black arrows. Adapted from McNagny and Hughes [20].

PODXL regulates epithelial cell motility predominantly through its interaction with the actin polymerisation complex, which is comprised of ezrin, a member of the ERM (*ezrin–radixin–moesin*) family, and the PDZ proteins NA+/H+ exchanger regulatory factor isoforms 1 and 2 (NHERF-1/2) (Figure 2) [13,89]. NHERF-1/2 and ezrin are adaptor proteins that have diverse binding partners and facilitate the interaction of PODXL with the cytoskeleton within epithelial cells. The interactions between PODXL, ezrin, and NHERF-1/2 have been reported to activate downstream intracellular signalling via the RhoA, Rac1, Cdc42, MAPK, and PI3K pathways to promote cancer metastasis [20].

In Madin–Darby canine kidney cells (MDCK), the PODXL-NHERF-1/2-ezrin complex activates RhoA and Rac1, two members of the Rho family of small guanosine triphosphatase (GTPases) [69]. Acting as signalling effectors, RhoA and Rac1 play a crucial role in regulating gene expression, cell polarity, proliferation, and locomotion [90,91]. Activation of these molecules initiates signalling through multiple pathways that can stimulate actin polymerisation and induce the formation of plasma membrane protrusions termed invadopodia [92]. Rac1 activation requires suppression of RhoGDI, a negative regulator of Rho GTPases, through ezrin as well as recruiting the Rho guanine nucleotide exchange factor 7 (ARHGEF7) through interacting with the first PDZ domain of NHERF-1 (Figure 2) [69]. Both these interactions are dependent on PODXL since the loss of PODXL abolishes these effects and decreases cell adhesion and migration [69,93]. 

In addition to Rac1, another Rho GTPase called Cdc42 is also associated with PODXL overexpression [93]. In Chinese hamster ovarian (CHO) cells, both Rac1 and Cdc42 activation parallels with PODXL-associated cell adhesion and migration [93]. Deletion of the first 49 amino acids within the N terminus of PODXL enhances cell adhesion and migration through increased Rac1 and Cdc42 activity [93]. This may explain how allelic imbalances and genetic mutations around PODXL may increase the risk of prostate cancer and tumour aggressiveness [16]. While deletion of the entire cytosolic tail of PODXL completely abolishes the effect of PODXL on cell adhesion and migration, deletion of just the terminal DTHL motif only reduces the effect [93]. This suggests that signalling pathways mediated by proteins other than NHERF-1/2 and ezrin also facilitate PODXL-induced cell migration. 

Cortactin, a prominent actin-binding protein, has been proposed as a potential candidate because it is found in the anti-PODXL immunoprecipitates of CHO cell lysates [93]. The DTHL motif of PODXL is further shown to regulate cortactin and Rac1/Cdc42 activation to enhance tumour metastasis and invadopodia formation [82]. These membrane protrusions have a matrix-degrading activity to aid migration and invasion during the metastatic process [94]. PODXL knockdown in breast cancer cell line MDA-MB231 markedly impairs invadopodia formation by suppressing the Rac1/Cdc42/cortactin signalling network, whereas overexpression of PODXL enhances invadopodia development and consequent metastasis [82]. Moreover, PODXL knockdown significantly impedes tumour dissemination and suppresses tumour colonisation [82]. Similarly, suppression of PODXL in oral squamous cancer cell lines SAS and FaDu effectively suppresses cortactin and F-actin colocalisation [83]. This is in conjunction with inhibition of focal adhesion kinase (FAK), a tyrosine kinase involved in cell signalling systems that promotes the development and progression of cancer [95]. Inhibition of FAK and its downstream molecule, paxillin, and suppression of cortactin and actin, reduces the formation of invadopodia. Furthermore, PODXL knockdown cells exhibit significantly diminished tumour migration, invasion, as well as proliferation and colony formation, indicating that PODXL expression is associated with tumour aggression through FAK/paxillin/cortactin signalling. 

PODXL overexpression in MCF-7 breast cancer cells, PC3 prostate cancer cells, as well as SGC-7901 gastric cancer cells, consistently enhances the mitogen-activated protein kinase (MAPK) and phosphatidylinositol 3-kinase (PI3K) signalling [13,78]. Constitutive activation of MAPK and PI3K pathways is known to trigger a cascade of responses in oncogenic transformation such as cell growth and proliferation, prosurvival pathways, epithelial–mesenchymal transition (EMT), and angiogenesis [96]. A well-established mechanism of PODXL action is through the induction of the metalloprotease (MMP) family [97]. MMPs are a family of proteolytic enzymes that degrade the extracellular matrix (ECM) and play an important role in tissue remodelling during cancer invasion and metastasis [98]. Indeed, PODXL overexpression correlates with increased MAPK and PI3K activity, as well as increased MMP-1 and MMP-9 secretion, in parallel with enhanced cell invasiveness of MCF7, PC3, and SGC-7901 cells [13]. In both MCF7 and PC3 cells, these effects are negated when ezrin is transiently knocked down, suggesting a dependent role of PODXL-ezrin interaction. In fact, PODXL expression alters the phosphorylation of ezrin at tyrosine residue 353 (Tyr^353^) and threonine residue 567 (Thr^567^). Phosphorylation of Tyr^353^ is required for ezrin to interact with PI3K, whereas phosphorylation of Thr^567^ activates ezrin to facilitate the formation of actin structures [99,100]. In SGC-7901 cells, however, the RUN and FYVE domain containing 1 (RUFY1) protein mediates the PODXL-induced effects in association with MAPK and PI3K signalling pathways [78]. RUFY1 is predominately localised to early endosomes and identified as a downstream effector of PI3K signalling to mediate effects of cell proliferation, colony formation, apoptosis, migration, and invasion [101]. Therefore, RUFY1 is a potential novel downstream target of PODXL. 

### 3.2. PODXL Mediates the Transforming Growth Factor β (TGFβ)-Induced EMT in Cancer

In order to metastasise and invade, epithelial cancer cells must first acquire a number of traits that allow them to lose cell-to-cell contact, become mesenchymal in morphology, and enter into the lumina of blood vessels where they can extravasate and proliferate within the microenvironment of a distant organ [102]. Type III EMT allows epithelial cells to acquire a mesenchymal cell-like phenotype which increases their migratory and invasive capacity [103]. The completion of the EMT process is indicated by degradation of the underlying basement membrane and the establishment of a motile mesenchymal cell that can invade and migrate away from the epithelium in which it originated. Under normal physiological conditions, EMT occurs during tissue repair and critical embryonic development to generate diverse cell types [104]. Once organ development is completed, the terminally differentiated cells often become immotile and can no longer go through further transformation [105]. It is thus not surprising that aberrant activation of EMT is often seen in pathological conditions such as cancer. As a key regulator of cell morphology, PODXL plays an important role in promoting EMT-like behaviour in cancer cells.

TGFβ is a transforming growth factor that enhances survival signalling pathways including PI3K/Akt and MAPK to drive EMT processes during tumour progression [106]. A role for PODXL in TGFβ-induced EMT has been reported in lung cancer metastases [81]. In lung adenocarcinoma cell line A549, PODXL is upregulated following TGFβ treatment in association with effective EMT transformation [63,81]. While cells overexpressing PODXL have a more mesenchymal morphology with the characteristic decrease of E-cadherin and increase of vimentin, cells with silenced PODXL expression exhibit a more epithelial morphology with decreased migration in association with an increase in E-cadherin and a reduction in vimentin [63,81]. Cell motility and migration are also increased in PODXL-overexpressed cells, as observed in aggressive lung adenocarcinomas [63]. In addition, PODXL expression is colocalised and suggested to interact with collagen I, a major component of the ECM, which is known to promote EMT via the formation of cell protrusions. PODXL has also been shown to interact with ezrin after TGFβ induction, suggesting that PODXL-ezrin interactions may also functionally participate in actin filament remodelling during the TGFβ-induced EMT process [107].

### 3.3. PODXL Facilitates EMT-Independent Tumour Budding

Although being regarded as an important paradigm, EMT is not obligatory for cancer metastasis. Other pathways, such as tumour budding which is a histological phenomenon characterised by the presence of small cohesive clusters of invasive and undifferentiated cancer cells (also known as tumour buds), are important [108]. While tumour budding has been described for decades, its importance as a potential prognostic marker of cancer has been recognised only recently.

**Figure 3:**
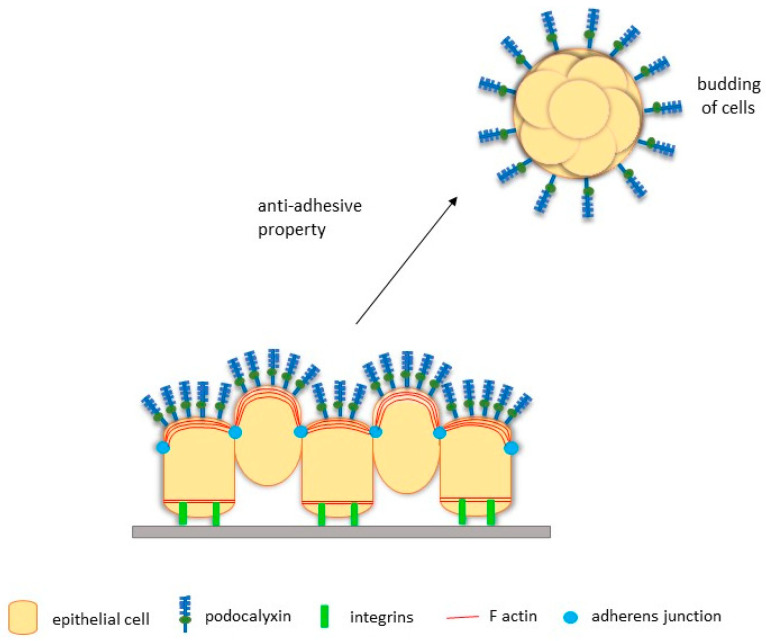
Proposed mechanisms of high levels of PODXL expression in cancer cells to promote tumour budding. Adapted from McNagny and Hughes [20].

PODXL is hypothesised to mediate tumour budding because its overexpression positively correlates with the lymphovascular invasion of breast cancer cells [74]. Subsequent studies with MCF-7 breast cancer cells show that overexpression of PODXL promotes the budding of cohesive nodules from the primary tumour in mice [109]. These nodules then migrate and invade the adjacent stromal tissue, demonstrating the ability of PODXL to induce collective tumour budding and subsequent invasion in vivo [109]. Additionally, PODXL overexpression promotes tumour spheroid formation in three-dimensional assays, consistent with the notion that PODXL promotes free-floating tumour clusters [110]. Formation of actin-rich lamellipodia protrusions is also observed on invading cell aggregates, which is likely associated with enhanced MAPK and PI3K activation, two pathways previously described as downstream of PODXL signalling [109]. Likewise, overexpression of PODXL in serous ovarian cancer cell line OVCAR3 decreases adhesion and increases the formation of small cohesive nodules, where PODXL is localised to the apical surface [14]. As serous tumour-like nodules are found in the abdominopelvic fluid of women with malignant growth, it is likely that the anti-adhesive property of PODXL may facilitate the initial detachment of nodules, leading to transperitoneal metastasis of serous cancers [14,111]. These findings in breast and ovarian cancer cells strongly suggest a potential role of PODXL in mediating tumour budding to induce cancer metastasis at distant sites (Figure 3).

### 3.4. PODXL Promotes Migration and Invasion of Cancer Cells

Cell migration in normal circumstances is a highly integrated process that enables embryonic morphogenesis, immune function, and tissue repair [112]. During cancer metastasis, cancer cells need to detach and cross extracellular matrix barriers. Cancer cells therefore must acquire abilities to migrate and interact with the surrounding ECM of the primary site to metastasise. Tumour cell motility into the surrounding tissue contributes significantly to the morbidity and mortality burden of cancer patients. PODXL is reported to mediate cancer cell migration and invasion.

Several studies have explored the metastatic mechanisms of PODXL in pancreatic ductal adenocarcinomas (PDACs). In addition to regulating molecular factors such as E-cadherin and vimentin to increase migratory potential, PODXL can bind to gelsolin, a cytoskeletal actin-severing protein that binds to actin, to promote cell motility and formation of lamellipodia protrusions in migrating cells [113]. In S2-013 and PANC-1 PADC cell lines, PODXL is colocalised and expressed in parallel with gelsolin [73]. Suppression of either PODXL or gelsolin results in a decrease in the formation of protrusions and inhibition of cell motility and invasion [73]. This indicates that PODXL and gelsolin may work cooperatively to induce the formation of protrusions in PDAC, thereby promoting motility and invasiveness of cells through their ability to increase actin-filaments via gelsolin-actin binding. However, a subsequent study found that, instead of altering actin dynamics in PDAC cells, PODXL knockdown impairs microtubule dynamics and focal adhesion [18]. Contrary to previous findings, ezrin was not identified in immunoprecipitated PODXL from SW1990 pancreatic cells, and PODXL knockdown failed to alter the activities of RhoA and Rac1, which are often induced by PODXL–ezrin binding; these results suggest that PODXL does not associate with ezrin in pancreatic cancer cells [18]. Instead, dynamin-2, a large GTPase protein involved in actin assembly, is suggested as a novel binding partner of PODXL [18]. Dynamin-2, when inhibited, decreases microtubule growth with similar efficiency as that of PODXL knockdown. Since dynamic instability of microtubules requires dynamin-2, microtubules may represent a downstream target of PODXL-dynamin-2 interactions that can facilitate PDAC cell migration and metastasis [114]. Moreover, PODXL knockdown leads to significant decreases in liver metastasis, compared to wild-type mice. PODXL is recently reported to promote metastasis in PDAC by capturing and activating the G-coupled protein complement receptor C5aR which is a critical cell motility inducer, that in turn recruits C5a, a soluble complement activation product involved in inflammation [85]. This PODXL-C5aR/C5a interaction, in turn, triggers PDAC invasion and metastasis by accelerating cellular motility. In AsPC-1 and MiaPaCa-2 PDAC cell lines, PODXL knockout leads to marked attenuation of C5aR, compared to the wildtype, implying that deficient PODXL may impair the protein stability of C5aR. Furthermore, mice injected with PODXL-knockout cells show significantly less liver and lymph nodal metastasis. Therefore, PODXL interactions with the C5aR/C5a axis appear to contribute to PDAC invasion and metastasis.

Members of the chloride intracellular channel (CLIC) family have been associated with cancer cell metastasis. In particular, CLIC5 is proposed as a risk marker for metastatic breast and rectal tumours [115,116,117]. Studies show that the interactions of PODXL with ezrin and CLIC5 are associated with invasiveness and migration of Huh7 hepatocellular carcinoma (HCC) cell lines [54]. PODXL, ezrin, and CLIC5 are colocalised in tumour regions of the rat liver, and PODXL and ezrin are present in immunoprecipitates of CLIC5. These findings suggest a scaffolding function of CLIC5, akin to that of NHERF-1/2. This is further supported by evidence that downregulation of CLIC5 significantly decreases the migration and invasion potential of HCC cells, likely in a PODXL–ezrin dependent manner [54]. In colon cancer, coexpression of the PDZ-binding motif (TAZ) with PODXL is associated with poor survival outcomes [86]. TAZ, as a key mediator of the Hippo signalling pathway, is important for normal organ homeostasis and tissue repair by regulating cell proliferation [118]. Overexpression of TAZ has been widely known to promote tumour metastasis and stemness properties in various malignancies [119,120]. Inhibition of PODXL in HCT15 colon cancer cells results in a downregulation of TAZ and its downstream targets, leading to a suppression of tumour invasion [86]. Therefore, PODXL may be an upstream regulator of the Hippo signalling pathway. 

In breast carcinoma models, tumour growth is significantly attenuated in mice injected with PODXL-knockdown cells, compared to controls which show significant cell proliferation [110]. Furthermore, tumours with deficient PODXL also exhibit impaired metastasis and significant reductions in the proportion of cells colonising a secondary site [110]. Similar findings have been made in gastric cancer (GC) cells, in which in vitro studies show significantly inhibited tumour growth and reduced liver metastasis in transfected mice, suggesting a functional role of PODXL in cancer metastasis [60]. Moreover, PODXL-knockout cells acquire epithelial phenotypes with upregulation of epithelial marker β-catenin and downregulation of MMP-2, resulting in reduced migration and invasion [60]. 

### 3.5. PODXL Mediates Extravasation during Metastasis via Binding to E- and L-Selectins, and Ezrin

Extravasation is a vital process that occurs once cells have undergone EMT and are dispersed from the primary tumour. As one of the last steps in cancer metastasis, extravasation involves dynamic interactions between cancer cells and the endothelium to establish a secondary metastasis [121].

Selectins are a family of transmembrane glycoproteins normally expressed by endothelial cells (E- and P-), leukocytes (L-), and platelets (P-) [122]. By directly binding tumour cells to the vascular endothelium, E-selectin, in particular, plays a pivotal role in cancer progression and metastasis [123,124]. PODXL expressed on LS174T colon cancer cells and various pancreatic carcinomas binds to both E- and L-selectins with high affinity [66,84]. PODXL is overexpressed by metastatic pancreatic cancer cell lines (Pa03C, Pa07C, and SW1990) but not by nonmalignant pancreatic counterparts [66]. PODXL knockdown in metastatic pancreatic cells significantly reduces their binding to immobilised E- and L-selectin under physiological flow conditions [66]. These results suggest a functional role of PODXL in facilitating tumour binding to selectin and metastasis.

As an important regulator of metastasis, ezrin and its association with PODXL has recently been explored in breast cancer extravasation [87,125]. In the highly metastatic breast cancer cell lines MDA-MB-231 and MDAMB-231, loss of PODXL reduces their ability to extravasate from in vitro endothelium, which is reversed by overexpression of PODXL [87]. Forced expression of PODXL in HMLER, a breast cancer cell line with no detectable PODXL, also markedly increases cell ability to extravasate, further confirming the importance of PODXL in extravasation [87]. Moreover, the intracellular domain of PODXL is shown to be important in extravasation. Ezrin, a well-known binding partner of PODXL that binds to its intracellular juxtamembrane domain, is suggested to critically mediate the role of PODXL in extravasation because knockdown of ezrin leads to loss of extravasation similar to that of PODXL knockout [87]. Furthermore, ezrin is colocalised with PODXL at the dorsal cortex of extravasating cells away from the endothelia, whereas cells without PODXL fail to redistribute ezrin, remain rounded in shape, and do not extravasate [87]. Therefore, extravasation in breast cancer cells appears to rely on PODXL-ezrin interactions, which likely enable cells to polarise and rearrange their cytoskeleton for transendothelial migration.

### 3.6. PODXL May Play an Important Role in Immune Evasion

Throughout cancer development and progression to metastasis, malignant cells tightly interact with the immune system where evading detection is an essential hallmark of successful metastasis [126]. Through releasing factors that promote tumour growth, metastasis, and angiogenesis, platelets play an important role in protecting tumour cells against the body’s immune system [127]. As PODXL is expressed by platelets in association with tumour cell–selectin interactions, PODXL has been speculated to confer mechanisms in tumour for immune evasion [66]. 

In MCF7 breast cancer cells, PODXL overexpression significantly inhibits T cell (CD4+ and CD8+) proliferation, supporting a previous study postulating an immunoregulatory role for PODXL in reducing T-lymphocyte proliferation in cardiac progenitor cells [17,128]. Natural killer (NK) cells are potent cytolytic lymphocytes of the innate immune system that plays a critical role in eliminating tumour cells [129], and evasion of NK immunosurveillance by strategies such as altering receptor–ligand activation patterns may promote tumour survival. While unaffecting NK cell proliferation, PODXL overexpression inhibits NK cell-activating receptors (e.g., NKG2D and CD16) and increases the expression of the classical MHC class I molecule (HLA-ABC), both of which protect cells from NK-mediated lysis [17]. In addition, PODXL overexpression activates CD16, a receptor involved in antibody-dependent cell cytotoxicity (ADCC), suggesting that PODXL may aid in tumour protection by impairing NK cell-mediated ADCC [17]. Furthermore, NK cells may acquire PODXL from cancer cells by trogocytosis, a mechanism of intercellular transfer of membrane fragments and molecules from tumour cells to NK cells during close contact to modify the phenotype and function of immune cells. Therefore, it is likely that the PODXL acquired by NK cells may inhibit normal cell-mediated cytotoxicity. Nevertheless, NK cell degranulation is not reduced, and PODXL has no effect on cytokine production, indicating a more intrinsic resistance of PODXL-expressing cancer cells to death [17]. Therefore, PODXL may act as an immunomodulatory molecule in cancer cells to suppress NK cell detection and evade immunosurveillance.

### 3.7. PODXL May Participate in Chemotherapy Resistance

The standard care for most cancers often includes surgery with additional chemotherapy and/or radiation therapy. However, metastasis poses clinical challenges since there are limited therapeutic options once cancer cells have established intrinsic/acquired chemoresistance to cancer drugs. The Discovery of novel targets may offer further insight into mechanisms underlying chemoresistance to shed light on new strategies to alleviate the problem.

Several studies have evaluated the effects of PODXL on conventional chemotherapy drugs. Colon cancer cells HT29 and HCT15, two cell lines that highly express PODXL, are resistant to the widely used colon cancer chemotherapy drugs 5-fluorouracil and irinotecan [86]. In contrast, HCT116 and LoVo cells, which express low levels of PODXL, are more sensitive [86]. More importantly, suppression of PODXL substantially reduces cancer cell viability when treated with chemotherapy [86].

In oral tongue squamous cell carcinoma (OTSCC) SCC-4 and Tca8113 cells, PODXL overexpression induces upregulation of the polycomb complex protein B lymphoma Mo-MLV insertion region 1 homolog (Bmi1) in a focal adhesion kinase (FAK) dependent manner, significantly increasing the IC50 of cisplatin and decreasing the cisplatin-induced apoptosis [88]. These effects are reversed by either knockdown of PODXL, overexpression of Bmi1, or introduction of a FAK inhibitor. Since Bmi1 may function as an epigenetic silencer of several genes, it may be involved in many cellular processes such as cell proliferation and apoptosis and has been associated with multiple human malignancies including OTSS [130]. Therefore, the cisplatin resistance induced by PODXL in OTSS is likely mediated by Bmi1/FAK-dependent pathways [88].

PODXL in promoting cancer cell proliferation and survival against chemotherapy and immunotherapy drugs has also be shown in nonepithelial cancers, such as astrocytoma, osteosarcoma, and mature B-cell lymphoma cells. In both astrocytoma and osteosarcoma, PODXL expression is associated with resistance to commonly used chemotherapy agents temozolomide and cisplatin, respectively, via a PI3K-dependent pathway [51,131]. Loss of PODXL sensitises these cells to these drugs for apoptosis and significantly decreases cancer cell viability [51,131]. In mature B-cell non-Hodgkin lymphoma cells, PODXL overexpression leads to higher resistance to corticosteroid medication dexamethasone, reactive oxygen species, and immunotherapy monoclonal antibody drug obinutuzumab [53]. Of note, these cancers are not of epithelial origin, and further studies are required to elucidate the role of PODXL in chemoresistance in epithelial cancers.

## 4. Conclusions

PODXL plays an important role in both normal tissue development and cancer progression. By predominantly regulating cell adhesion and morphology, PODXL exerts important functions throughout the body including optimal glomerular function. On the other hand, PODXL is aberrantly expressed in several cancers, many of which are originated from the epithelium, and PODXL is upregulated in highly aggressive and malignant cancers. PODXL overexpression is associated with cancer hallmarks such as EMT, metastasis, invasion, survival, and resistance to drugs, all of which promote tumour aggression and poor prognosis. While the detailed molecular mechanisms by which PODXL promotes tumourigenesis remain to be elucidated, PODXL is proposed to interact with several proteins and downstream signalling pathways that are critical in promoting cancer metastasis and subsequent invasion. Moreover, PODXL has been implicated in facilitating immune evasion and resistance of cancer cells to chemotherapy drugs. Therefore, PODXL may be a valuable diagnostic and prognostic biomarker and a potential therapeutic target for managing malignant epithelial cancers.

## Figures and Tables

**Table 1:** Summary of PODXL expression profiles in epithelial cancers.

Types of Carcinoma	PODXL Expression	Diagnostic and Prognostic Significance	References
Breast	Overexpressed in a subset of invasive breast carcinomas (6%), and associated with eightfold increase in relative risks of poor outcome (RR: 8.4). Overexpression is predominantly in higher grade, estrogen and progesterone negative, and lymphatic invasive tumours.	Predictor of cancer progression/poor prognosis. Correlated with increased risk of aggressive phenotype, hormone receptor negativity, and high grade	[15,54]
Colorectal	Overexpressed in 5–13% of patients. Associated with increased relative risks of death (HR: 1.98–2.0), reduced 5-year overall survival rates (HR: 1.85–2.28), shorter time to recurrence (HR: 2.11–2.93), and disease-free survival (HR: 2.44). Positive expression is also highly concordant between primary and metastatic lesions.	Predictor of metastatic disease and poor prognosis (in subgroups of left hemi-colon and rectum).	[19,55,56,57]
Gastric	Positive expression in 36–75% of patients, correlating with advanced tumour stage and metastasis, poor tumour differentiation, reduced time to recurrence (45% vs. 88%), and disease-specific 5-year survival (24% vs. 43%) as well as overall survival rates (40% vs. 55%).	Predictor of metastatic disease, tumour stage, poor differentiation, and prognosis.	[58,59,60,78]
Liver (Hepatocellular carcinoma HCC)	Positive expression in 78% of cases. High expression in sinusoidal endothelia and tumour-like lesions but not in normal adjacent tissues.	Diagnostic marker	[54,61]
Lung			
Small cell lung carcinoma	Positive expression in 87% of cases.	Diagnostic marker	[62]
Lung adenocarcinoma	Positive expression only in invasive lung adenocarcinomas.	Predictor of aggressive and invasive tumours	[63]
Esophageal	Positive expression in 85% of patients, associated with shorter time to recurrence (35% vs. 75%) and 5-year overall survival (28% vs. 69%).	Predictor of poor prognosis	[59]
Oral squamous cell	Positive expression in 68% of cases	Diagnostic marker to differentiate oral squamous cell carcinoma from other oral cancers	[64]
Ovarian	Overexpressed in 87% of high-grade serous carcinomas. Cell surface expression associated with decreases in disease-free survival (HR: 1.73)	Predictor of poor prognosis in high-grade serous subgroup	[14]
Pancreatic ductal adenocarcinoma (PDAC)	Positive expression in 34–44% and overexpressed in 29.4% cases of PDAC cases, associated with high-grade tumours. High expression is associated with poor differentiation, perineural and perivascular invasion, and increased relative risks of death (HR: 1.62–2.21). Overexpression of PODXL in combination with ITGB1 associates with poor postoperative outcomes.	Predictor of high-grade tumours, aggressive phenotype and poor prognosis	[65,66,67,68,73,79]
Periampullary	Positive PODXL expression in 46% of pancreatobillary- type (PB-type) subgroup, associated with female sex and poor differentiation grade. Positive PODXL expression in 18% of intestinal-type (I-type); associated with reduced recurrence free survival (HR: 2.44) and overall survival (HR: 2.32)	Predictor of poor differentiation in PB-type subgroup. Predictor of aggressive phenotype and poor prognosis in I-type subgroup.	[67]
Prostate	PODXL germ-line mutation is associated with increased risks of developing aggressive prostate cancer.	Genetic marker	[16]
Renal	Overexpressed in a subset of patients (9.6%), associated with reduced rates of metastasis-free survival (HR: 3.59) and disease-specific survival (HR: 7.46).	Predictor of aggressive phenotype, metastatic disease, and poor prognosis	[69]
Thyroid	Identified only in undifferentiated thyroid carcinoma (UTC), and positively expressed in 52% of UTC cases.	Diagnostic marker for UTC	[49]
Urothelial (bladder)	Positive expression associated with higher-grade tumours, reduced 5-years of overall survival (HR: 2.05–3.28), disease-specific survival (HR: 2.7), and 2-year progression-free survival (HR: 7.16).	Predictor of high-grade tumours and poor prognosis	[70,71]
Uterine endometrioid adenocarcinoma (EA)	Positive expression detected in 36% of uterine EA cases and associated with higher tumour grades.	Predictor of high-grade tumours	[72]

**Table 2:** Proposed PODXL mechanisms in cancer metastasis.

Mechanism	Proteins That PODXL Interacts with	Cancer Type
EMT	Colocalisation and interaction with collagen I, interaction with ezrin, E-cadherin, and vimentin	Lung [63,81]
Migration and Invasion	Colocalisation and interaction with gelsolin	Pancreatic [73]
	Binding to ezrin	Breast [13]
	Enhancing the activation of PI3K, Ras/rac1, and MAPK signalling pathway	Pancreatic [18]
	Activation of rac1/cdc42/cortactin signalling	Breast [82]
	Activation of FAK	Oral squamous cell [83]
	Increasing MMP	Breast [13]
	Binding to selectins (E- and L-)	Colon [84]Pancreatic [66]
	Colocalisation and interaction with CLIC5	Liver [54]
	Binding to dynamin-2	Pancreatic [18]
	Stimulating the C5aR/C5a axis	Pancreatic [85]
	Associates with TAZ	Colon [86]
Extravasation from the vasculature	Binding to ezrin	Breast [87]
Immune evasion	Inhibiting NK cell receptors Increasing the expression of MHC I molecule (HLA-ABC)	Breast [17]
Chemoresistance	Increasing Bmi1	Oral tongue squamous cell [88]
